# Antibody-Based Therapeutics in Autoimmune Diseases: Mechanisms, Challenges, and Future Perspectives

**DOI:** 10.3390/ijms27188030

**Published:** 2026-09-09

**Authors:** Naim Mahroum

**Affiliations:** International School of Medicine, Istanbul Medipol University, Kavacık, Göztepe Mah, Atatürk Cd. No: 40, Beykoz, 34810 Istanbul, Türkiye; naim.mahroum@gmail.com; Tel.: +90-216-681-51-00; Fax: +90-212-531-75-55

**Keywords:** autoimmunity, immunomodulation, immunosuppressants, antibody-based treatment, monoclonal antibodies

## Abstract

Autoimmune diseases encompass nearly 100 distinct conditions characterized by dysregulated immune responses against self-antigens, resulting in tissue damage, chronic inflammation, and substantial morbidity. Over recent decades, the global burden of autoimmune diseases has increased, while their therapeutic management has undergone a paradigm shift from broad, nonspecific immunosuppression toward targeted biologic therapies. Among these, antibody-based therapeutics, including monoclonal antibodies targeting cytokines, cell-surface molecules, and co-stimulatory pathways, have transformed the treatment of numerous systemic and organ-specific autoimmune disorders. Nevertheless, important challenges remain, including primary non-response, secondary loss of efficacy related to immunogenicity or disease adaptation, increased susceptibility to infections, and mechanistic redundancy among therapeutic targets. This review provides a comprehensive overview of the cellular and molecular mechanisms underlying antibody-based therapies, summarizes major therapeutic target classes across autoimmune diseases, and examines their clinical and mechanistic limitations. Emerging therapeutic platforms, including bispecific antibodies, antibody–drug conjugates (ADCs), novel Fc-engineering, and cellular approaches such as CD19-targeted chimeric antigen receptor (CAR)-T therapy, are also discussed. Finally, antigen-specific immunotherapies designed to restore antigen-specific immune tolerance while preserving systemic host defense are explored. These approaches may represent an important next frontier in the treatment of autoimmune diseases, moving beyond broad systemic immune modulation toward more selective and durable immune control.

## 1. Introduction

Autoimmune diseases represent a diverse group of chronic conditions characterized by dysregulated immune responses against self-antigens, leading to tissue damage of various degrees, and are associated with significant morbidity and mortality [1]. These disorders share diverse etiopathogenetic mechanisms, which may potentially serve as a basis for therapeutic strategies [2]. In this review, the term autoimmune diseases is used broadly to encompass clinically relevant immune-mediated inflammatory disorders in which antibody-based immune modulation has an established or emerging therapeutic role. Historically, systemic autoimmune disorders were managed primarily through broad-spectrum immunosuppressive agents, such as systemic corticosteroids, antimetabolites, and calcineurin inhibitors. While effective in controlling acute inflammatory activity, these non-targeted regimens are associated with substantial cumulative toxicities with prolonged use, including metabolic disorders, organ damage, and increased susceptibility to infections [3].

Over the past two decades, the therapeutic landscape of autoimmune diseases has been transformed by the advent of antibody-based therapies, which have enabled targeted modulation of key immune pathways. These biologic agents, including monoclonal antibodies (mAbs) directed against cytokines, immune cell surface markers, and co-stimulatory molecules, have significantly improved clinical outcomes in multiple autoimmune conditions [4]. The introduction of mAbs has progressively shifted the therapeutic paradigm from broad, non-specific immunosuppression toward targeted modulation of specific pathogenic pathways [5].

Despite these transformative advances, substantial challenges remain. A considerable proportion of patients experience primary non-response or develop secondary treatment failure due to anti-drug antibody formation, pathway compensation, or mechanistic redundancy [6]. Additionally, targeting shared immune elements carries risks of opportunistic infections and potential long-term safety concerns [7]. This review provides a broad overview of mechanisms, challenges, and future perspectives of antibody-based therapeutics, with a brief discussion of antigen-specific approaches.

## 2. The Role of Antibodies in Autoimmune Diseases

Antibodies are central mediators of adaptive immunity, providing protection against invading pathogens while maintaining immune homeostasis. In autoimmune diseases, however, the mechanisms that preserve self-tolerance become disrupted, leading to the generation of autoreactive antibodies that recognize self-antigens and perpetuate chronic inflammation [8]. Beyond their direct pathogenic effects, antibodies interact with multiple immune cell populations, complement pathways, and inflammatory mediators, making them both key drivers of disease and attractive therapeutic targets. In addition to B cells, autoreactive T-cell subsets, particularly T follicular helper (Tfh) and Th17 cells, play a pivotal role in driving disease pathogenesis by orchestrating autoantibody production and mediating direct tissue injury, thereby serving as critical upstream targets for therapeutic intervention [9]. Understanding the physiological and pathological functions of antibodies therefore provides the biological foundation for the development of antibody-based therapies.

### 2.1. Protective vs. Pathogenic Immunity

Although antibodies are indispensable for host defense, their protective functions rely on tightly regulated mechanisms of central and peripheral immune tolerance that eliminate or silence autoreactive lymphocytes. Breakdown of these tolerance checkpoints transforms protective humoral immunity into chronic autoimmunity, providing the biological rationale for therapeutic strategies aimed at selectively restoring immune balance rather than indiscriminately suppressing immune function [10].

Under physiological conditions, humoral immunity depends on tight immune checkpoint control, somatic hypermutation regulation, and central/peripheral B-cell tolerance mechanisms [11]. The breakdown of self-tolerance driven by environmental triggers, genetic susceptibility, and cytokine imbalance leads to the escape of autoreactive B cells [12]. B-cell dysregulation manifests not only in autoantibody production, but also in hyperactive antigen presentation to T cells and excessive pro-inflammatory cytokine secretion (e.g., IL-6, TNF-α). Re-establishing control over these autoreactive pathways provides the physiological rationalization for targeted antibody-based therapeutics.

### 2.2. Pathogenic Autoantibodies

Autoantibodies contribute to disease through several complementary mechanisms that extend beyond simple antigen recognition. Depending on their specificity and tissue distribution, they may directly damage target cells, alter receptor function, activate complement, or promote the formation of pathogenic immune complexes, collectively amplifying inflammation and tissue injury [8].

Autoantibodies contribute directly to tissue destruction through distinct immunological pathways:

- **Direct tissue and receptor targeting:** Autoantibodies bind directly to self-antigens expressed on cell surfaces or extracellular matrix elements, inducing direct cell death or functional alterations (e.g., anti-acetylcholine receptor antibodies in myasthenia gravis, or anti-desmoglein antibodies in pemphigus vulgaris) [13].
- **Immune complex formation:** Soluble autoantibodies interact with systemic circulating autoantigens to form immune complexes (ICs). These complexes deposit in vascular microenvironments, particularly within renal glomeruli, synovial membranes, and dermal capillaries, driving immune-complex-mediated vasculitis and glomerulonephritis (e.g., systemic lupus erythematosus).
- **Complement activation:** Bound autoantibodies trigger the classical complement cascade via C1q engagement, generating anaphylatoxins (C3a, C5a), recruiting neutrophils, and forming the membrane attack complex (MAC, C5b-9) to mediate lytic cellular destruction.

## 3. Mechanisms of Action of Antibody-Based Therapeutics

**Figure 1:**
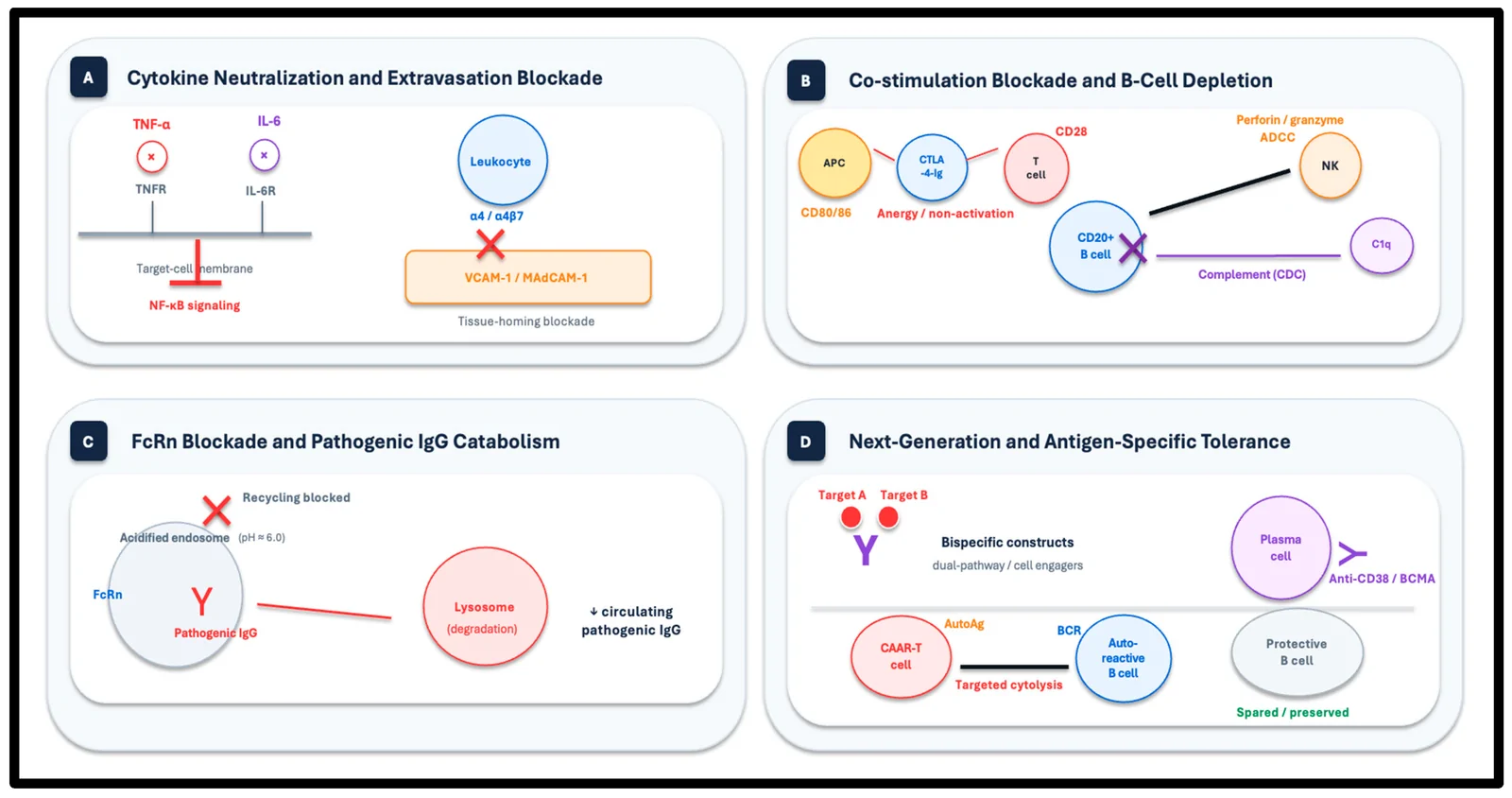
Mechanistic framework of antibody-based therapeutics in autoimmune diseases. (**A**) Cytokine neutralization and extravasation blockade: mAbs neutralize pro-inflammatory cytokines (e.g., TNF-α, IL-6), while integrin inhibitors (e.g., anti-α4, anti-α4β7) block leukocyte-endothelial interactions (VCAM-1, MAdCAM-1) to halt tissue homing. (**B**) Co-stimulation blockade and B-cell depletion: CTLA-4-Ig blocks CD80/CD86-CD28 signaling to induce T-cell anergy. Anti-CD20 mAbs deplete B cells via NK-cell-mediated ADCC and complement-mediated lysis (CDC). (**C**) FcRn blockade and IgG catabolism: FcRn inhibitors block endosomal IgG recycling, directing pathogenic autoantibodies to lysosomes for degradation. (**D**) Next-generation platforms and antigen-specific tolerance: Bispecific constructs enable dual targeting; plasma-cell-directed agents (anti-CD38, anti-BCMA) clear long-lived antibody secretors; and CAAR-T cells selectively lyse autoreactive B cells while preserving protective humoral immunity. Abbreviations: ADCC, antibody-dependent cellular cytotoxicity; APC, antigen-presenting cell; AutoAg, autoantigen; BCMA, B-cell maturation antigen; BCR, B-cell receptor; CAAR, chimeric autoantibody receptor; CDC, complement-dependent cytotoxicity; CTLA-4, cytotoxic T-lymphocyte-associated protein 4; FcRn, neonatal Fc receptor; Ig, immunoglobulin; IL-6(R), interleukin-6 (receptor); MAdCAM-1, mucosal vascular addressin cell adhesion molecule 1; NF-κB, nuclear factor kappa B; NK, natural killer; TNF-α, tumor necrosis factor alpha; TNFR, TNF receptor; VCAM-1, vascular cell adhesion molecule 1.

Therapeutic antibody-based agents have transformed the management of autoimmune diseases by enabling selective modulation of critical immune pathways. Unlike conventional immunosuppressive agents, which broadly inhibit immune activity, antibody-based therapies are designed to interfere with specific molecular or cellular targets involved in disease pathogenesis [4]. Depending on their mechanism of action, these biologic agents neutralize inflammatory cytokines, deplete pathogenic immune-cell populations, inhibit lymphocyte activation, or prevent inflammatory cell trafficking into affected tissues (Figure 1). In this review, the term “antibody-based therapeutics” is used as an umbrella term encompassing monoclonal antibodies (mAbs) and, where mechanistically relevant, antibody-derived molecules and Fc-containing fusion proteins.

### 3.1. Cytokine Neutralization

Cytokines orchestrate immune-cell communication and regulate the magnitude, duration, and resolution of inflammatory responses. Dysregulated cytokine production is a hallmark of many autoimmune diseases, making these soluble mediators among the earliest and most successful therapeutic targets for antibody-based therapy [14].

Cytokine-targeted antibody-based agents selectively bind soluble or membrane-bound inflammatory cytokines, blocking their interactions with cognate cell-surface receptors:

- **Anti-TNF agents** (infliximab, adalimumab, etanercept, certolizumab pegol, golimumab): Neutralize soluble and transmembrane TNF, preventing its binding to TNF receptor 1/2 (TNFR1/TNFR2) and thereby attenuating downstream nuclear factor kappa B (NF-κB)-dependent pro-inflammatory gene expression. Subsequently, this action results in reducing macrophage activation, chemokine production, and joint/tissue erosion [15].
- **Anti-IL-6/IL-6R agents** (tocilizumab, sarilumab): Interrupt acute-phase reactant synthesis, systemic inflammation, and T-helper 17 (Th17) cell differentiation [16].
- **Anti-IL-17 and Anti-IL-12/23 agents** (secukinumab, ixekizumab, ustekinumab, risankizumab): Disrupt the IL-23/Th17 axis, pivotal in mucosal and cutaneous chronic autoimmune pathologies [17].

### 3.2. Cell Depletion Strategies

In many autoimmune disorders, pathogenic immune-cell populations sustain chronic inflammation through antigen presentation, cytokine secretion, and autoantibody production. Selective depletion of these cellular subsets interrupts disease propagation while preserving much of the remaining immune repertoire and host defense [18].

Depleting specific immune subsets eliminates pathogenic cellular pools. Anti-CD20 mAbs (e.g., rituximab, ocrelizumab, ofatumumab) target pre-B and mature B lymphocytes. Binding to CD20 induces rapid cell elimination via programmed cell death, complement activation, and cellular phagocytosis. Because CD20 is not expressed on early hematopoietic stem cells or fully differentiated long-lived plasma cells, B-cell depletion allows subsequent repopulation while generally sparing established long-lived plasma-cell populations [18,19].

### 3.3. Immune Checkpoint Modulation and Co-Stimulation Blockade

Immune checkpoint inhibitors (ICIs), including antibodies targeting CTLA-4 and the PD-1/PD-L1 axis, enhance T-cell activation by removing inhibitory signals that normally maintain peripheral immune tolerance. Although this principle has revolutionized cancer therapy, the development of immune-related adverse events and the emergence or exacerbation of autoimmune diseases following ICI treatment have highlighted the critical role of these pathways in restraining autoreactive immunity [20]. Conversely, therapeutic strategies that enhance inhibitory signaling or block co-stimulatory pathways can suppress autoreactive T-cell activation while preserving broader immune function. For example, Abatacept (a CTLA-4-Ig fusion protein) binds to CD80 and CD86 on antigen-presenting cells (APCs), selectively blocking the CD28 co-stimulatory signal required for full CD4+ T-cell activation and proliferation [21].

### 3.4. Inhibition of Immune Cell Trafficking

Autoimmune tissue injury depends not only on immune-cell activation but also on the recruitment and migration of inflammatory leukocytes into target organs [22]. Preventing immune-cell trafficking therefore represents an alternative therapeutic strategy that limits tissue inflammation without directly suppressing immune-cell function.

Adhesion molecule inhibitors prevent autoreactive lymphocytes from extravasating into target tissues. Integrin blockers such as natalizumab (anti-α4-integrin) and vedolizumab (anti-α4β7 integrin) prevent lymphocyte trafficking across the blood–brain barrier and intestinal gut mucosa, respectively [23].

### 3.5. Fc-Mediated Effector Mechanisms

Beyond target neutralization, the engineered Fc domains of therapeutic monoclonal antibodies recruit host immune machinery to eliminate target cells. These Fc-mediated interactions substantially enhance therapeutic efficacy by promoting selective elimination of pathogenic immune cells through antibody-dependent cellular cytotoxicity (ADCC), complement-dependent cytotoxicity (CDC), and other Fc receptor-mediated pathways [24]. Beyond simple antigen neutralization, the Fc domain of IgG-based therapeutics recruits host immune machinery:

- **Antibody-dependent cellular cytotoxicity (ADCC):** The Fc region binds FcγRIIIa (CD16) on natural killer (NK) cells, triggering perforin/granzyme release and target cell lysis [24].
- **Complement-dependent cytotoxicity (CDC):** Binding of C1q to the clustered Fc portions of bound antibodies initiates the complement cascade, culminating in cell membrane lysis [25].

## 4. Selected Clinically Established Antibody-Based Therapies

Advances in our understanding of autoimmune pathogenesis have led to the development of numerous antibody-based therapeutics targeting distinct components of the immune response. These biologic agents differ in their molecular targets, mechanisms of action, and approved clinical indications, reflecting the considerable biological heterogeneity of autoimmune diseases. Table 1 summarizes selected major classes of antibody-based therapeutics, their principal molecular targets, representative clinical indications, and mechanisms of action.

## 5. Clinical Challenges and Limitations

Despite their remarkable clinical success, antibody-based biologics are not universally effective and remain associated with several important limitations. Variability in treatment response, immunogenicity, infectious complications, safety concerns, and economic barriers continue to influence long-term patient outcomes and represent major challenges in the management of autoimmune diseases [7].

### 5.1. Primary and Secondary Non-Response

Clinical response to biologic therapies varies considerably. Primary non-response occurs when patients fail to achieve clinical endpoints from therapy onset, often reflecting underlying disease heterogeneity where the targeted pathway is not the primary driver of inflammation. Secondary non-response involves initial treatment efficacy followed by loss of response over time, frequently driven by immunogenicity and the development of anti-drug antibodies (ADAs) [26]. ADAs can neutralize therapeutic binding sites or accelerate systemic clearance [27,28].

### 5.2. Safety Concerns

Although selective immune modulation generally offers a more favorable safety profile than conventional immunosuppression, interference with critical immune pathways inevitably carries risks that require careful patient selection, screening, and long-term monitoring.

Suppression of targeted inflammatory pathways carries inherent safety risks:

- **Infectious vulnerability:** TNF inhibition markedly increases the risk of reactivating latent *Mycobacterium tuberculosis* and opportunistic fungal infections [29]. Targeted B-cell depletion predisposes patients to severe viral infections and viral reactivation.
- **Malignancy and paradoxical reactions:** Although the long-term malignancy risks associated with biologic therapies remain an area of ongoing evaluation, continued clinical monitoring is warranted [30]. Furthermore, paradoxical inflammatory reactions, such as new-onset psoriasis following anti-TNF therapy, represent clinically important treatment-related complications [31].

### 5.3. Cost, Accessibility, and Loss of Precision

Beyond biological limitations, the widespread implementation of antibody-based therapies is constrained by substantial economic and practical challenges where biologic therapeutics incur substantial financial costs, limiting global access [32]. Moreover, current clinical administration often relies on trial-and-error paradigms (“loss of precision”), where different patients with identical diagnostic labels receive the same agent despite distinct underlying immunophenotypes [33]. Subsequently, treatment selection often remains largely empirical, reflecting the current inability to consistently match individual patients with the biologic agent most appropriate for their underlying disease mechanisms.

## 6. Mechanistic Challenges

Many of the clinical limitations encountered with antibody-based therapeutics stem directly from the mechanistic interplay between single-target pharmacodynamics and the intricate biological network of autoimmune diseases. Because these agents operate through discrete, target-specific mechanisms such as selective cytokine neutralization or cell-surface marker engagement, their long-term efficacy is frequently constrained by compensatory signaling pathways, persistent autoreactive cellular niches, and restricted tissue penetrance. Consequently, incomplete or transient therapeutic responses often reflect underlying mechanistic redundancy and compartmentalization rather than an inherent drug defects [34]. These core mechanistic challenges encompass several key aspects:

- **Pathway redundancy:** Autoimmunity is rarely driven by a single cytokine or pathway. Neutralizing one axis (e.g., IL-6) often prompts compensatory up-regulation of alternative inflammatory pathways [34].
- **Incomplete B-cell and plasma cell targeting:** Standard anti-CD20 therapies effectively deplete circulating CD20-expressing B cells but spare CD20-negative long-lived plasma cells residing within protective bone marrow niches. These cells may continue to secrete pathogenic autoantibodies despite peripheral B-cell depletion [35].
- **Tissue compartmentalization:** Circulating therapeutic antibodies often exhibit poor penetration into specialized tissue microenvironments, such as dense synovial tissues, mucosal niches, or across the blood–brain barrier [36].

## 7. Emerging and Next-Generation Antibody-Based Strategies

The limitations of conventional antibody-based therapies have prompted the development of next-generation platforms designed to achieve greater biological precision, overcome pathway redundancy, and selectively eliminate pathogenic immune-cell populations. Rather than targeting a single inflammatory mediator, these approaches seek to intervene at multiple levels of immune dysregulation, including simultaneous blockade of complementary pathways, targeted intracellular delivery of therapeutic payloads, modulation of antibody pharmacokinetics, and direct depletion of long-lived autoreactive plasma cells. Although several of these strategies remain investigational in autoimmune diseases, they illustrate an important shift toward more selective and mechanistically tailored immunomodulation.

### 7.1. Bispecific Antibodies (bsAbs)

Bispecific antibodies simultaneously engage two distinct antigens, thereby enabling coordinated modulation of two biological pathways or the physical bridging of different cell populations [37]. In autoimmune diseases, dual-targeting constructs may overcome biological redundancy that limits the efficacy of single-target therapies. A representative example is dual inhibition of IL-17A and IL-17F, as achieved by bimekizumab, which targets two closely related cytokines that contribute to persistent inflammatory signaling. Its clinical efficacy has been demonstrated across several immune-mediated inflammatory diseases, including psoriatic arthritis and axial spondyloarthritis, in the phase 3 BE OPTIMAL, BE MOBILE, and BE VITAL programs, supporting the therapeutic rationale for simultaneous IL-17A/F blockade [38,39,40]. Beyond simultaneous cytokine blockade, bispecific antibodies can be designed as T-cell or NK-cell engagers, redirecting cytotoxic effector cells toward pathogenic B cells or other disease-driving immune-cell populations. Such designs may therefore provide a means of achieving deeper and more selective immune-cell depletion than conventional mAbs [37].

### 7.2. Antibody–Drug Conjugates (ADCs)

Antibody–drug conjugates (ADCs) combine the target specificity of mAbs with a pharmacologically active payload linked through a specialized linker. Following binding to a selected cell-surface antigen and internalization, the payload can be released intracellularly, allowing potent cytotoxic or immunomodulatory agents to be delivered preferentially to pathogenic cells. In autoimmune diseases, this approach could potentially enable selective depletion or functional modulation of disease-driving immune-cell populations while reducing systemic exposure to the therapeutic payload. Experimental autoimmune applications have explored this principle using immune-cell-directed antibodies linked to glucocorticoids or other pharmacologically active payloads. For example, glucocorticoid-conjugated anti-CD20 constructs have been investigated as a means of delivering corticosteroid activity selectively to B cells, with the aim of retaining B-cell-directed immunomodulation while reducing the systemic adverse effects associated with conventional glucocorticoid therapy [41]. Similarly, antibody–drug conjugate strategies targeting CD11a have been explored to deliver immunomodulatory payloads to activated leukocyte populations and thereby enhance cellular selectivity [42,43]. However, unlike their established role in oncology, ADCs remain an emerging and largely investigational strategy in autoimmune and rheumatic diseases, with ongoing studies exploring appropriate targets and safety profiles [44]. Their development is therefore dependent on identifying disease-relevant cellular targets, optimizing payload and linker properties, and demonstrating that selective delivery provides a meaningful therapeutic advantage over conventional immunosuppression.

### 7.3. Fc Engineering and FcRn-Directed Therapies

Modifying the Fc domain of antibodies provides an additional means of controlling their pharmacokinetic and immunological properties, including serum half-life, Fc receptor interactions, and effector functions. Engineering antibody–Fc interactions can therefore be used either to prolong therapeutic exposure or, conversely, to enhance the clearance of pathogenic immunoglobulins. A clinically important example is targeting the neonatal Fc receptor (FcRn), which normally protects IgG from lysosomal degradation and thereby prolongs its circulating half-life. FcRn-blocking agents such as efgartigimod accelerate the catabolism of endogenous IgG, including pathogenic autoantibodies, providing a targeted strategy for antibody-mediated autoimmune diseases and reducing reliance on approaches such as plasmapheresis. In the phase 3 ADAPT trial, efgartigimod demonstrated clinical efficacy in generalized myasthenia gravis, supporting FcRn inhibition as a clinically relevant mechanism for selectively reducing pathogenic IgG [45].

### 7.4. Targeting Plasma Cells

A major limitation of conventional B-cell depletion is the persistence of long-lived plasma cells, which can survive despite effective depletion of circulating CD20-positive B cells and continue producing pathogenic autoantibodies. Consequently, increasing attention has focused on plasma-cell-associated targets such as CD38 and B-cell maturation antigen (BCMA). Anti-CD38 antibodies, particularly daratumumab, can target antibody-secreting plasma cells and plasmablasts and have shown early clinical and serological activity in refractory autoimmune diseases, while BCMA represents an additional target because of its preferential expression on antibody-secreting plasma-cell populations [35]. These approaches may therefore complement conventional B-cell-directed therapies by targeting the persistent cellular reservoirs responsible for sustained autoantibody production. Nevertheless, plasma-cell-directed antibody therapies remain at an earlier stage of development in autoimmunity, and their long-term efficacy, safety, and effects on protective humoral immunity require further investigation.

## 8. Antigen-Specific and Immune-Resetting Therapies

In parallel with the development of increasingly selective antibody- and cell-based therapeutics, strategies aimed at actively restoring immune tolerance are gaining increasing attention. Antigen-specific immunotherapies seek to selectively silence or eliminate autoreactive immune responses while preserving protective immunity against unrelated antigens [46] (Figure 1D). In contrast, immune-resetting approaches such as CD19-targeted CAR-T therapy do not require identification of a single autoantigen but instead seek to eliminate broad autoreactive B-cell compartments and permit subsequent immune reconstitution. Thus, these emerging strategies represent two complementary approaches to disease modification: antigen-specific tolerance induction and deep cellular immune resetting. Unlike conventional immunosuppressive approaches, their ultimate objective is not simply to suppress inflammation but to re-establish antigen-specific immune tolerance and potentially achieve durable, treatment-free disease control. Although most approaches remain experimental or in early clinical development, advances in autoantigen identification, tolerogenic vaccine design, cellular engineering, and targeted delivery systems are bringing antigen-specific therapy closer to clinical translation [46,47].

Table 2 summarizes the key differences in antigen-specific therapies in terms of their effects on systemic host defense, target range, potential risk of opportunistic infection, and therapeutic objectives.

Antigen-specific approaches include:

- **Peptide immunotherapy and tolerogenic vaccines:** Delivery of disease-associated autoantigenic epitopes in a non-inflammatory or tolerogenic context can promote peripheral tolerance through mechanisms including T-cell anergy, deletion of autoreactive clones, and expansion or induction of antigen-specific regulatory T cells. Tolerogenic nanoparticles, modified peptides, and tolerogenic dendritic-cell approaches are being investigated as means of delivering self-antigens while avoiding the inflammatory signals that would otherwise reinforce autoimmunity [46,47].
- **Chimeric autoantibody receptor (CAAR) T-cells:** CAAR-T cells represent a highly selective cellular strategy in which the extracellular domain of the receptor incorporates the relevant autoantigen. This enables engineered T cells to recognize and eliminate autoreactive B cells through their antigen-specific surface B-cell receptors, potentially sparing the broader pool of healthy B cells. CAAR-T approaches targeting disease-associated autoantibodies, including strategies directed against desmoglein 3 in pemphigus vulgaris and muscle-specific kinase (MuSK) in myasthenia gravis, illustrate the potential of antigen-specific cellular depletion [48]. These approaches remain investigational, with antigen selection, durability, safety, and manufacturing complexity representing important translational challenges.
- **CD19-targeted CAR-T cells and immune resetting:** In contrast to CAAR-T cells, which are designed to recognize disease-associated autoreactive B cells through their antigen-specific B-cell receptors, CD19-targeted CAR-T cells induce broad depletion of the CD19-positive B-cell compartment and may therefore achieve a more profound reset of B-cell immunity. Early clinical studies in severe, refractory autoimmune diseases have demonstrated marked clinical improvement and drug-free remission, accompanied by profound B-cell depletion followed by immune reconstitution in patients treated with CD19 CAR-T cells [49]. Subsequent studies have extended these observations across systemic autoimmune diseases, including systemic lupus erythematosus, systemic sclerosis, and idiopathic inflammatory myopathies, suggesting that CAR-T therapy may reset autoreactive B-cell immunity more deeply than conventional B-cell depletion [50]. Unlike anti-CD20 mAbs such as rituximab, which spare most long-lived plasma cells, CD19 CAR-T may achieve deeper B-cell-lineage depletion and more substantial reduction in pathogenic autoantibody production. However, its greater therapeutic intensity, including the need for lymphodepletion and specialized cellular-therapy infrastructure, as well as risks such as cytokine-release syndrome and hypogammaglobulinemia, currently limit its use to highly selected refractory patients.

Despite their precision, antigen-specific therapies face several major hurdles. These include accurately identifying the pathogenic autoantigen in diseases characterized by extensive antigenic heterogeneity and epitope spreading, determining the optimal antigenic epitope and therapeutic modality, accounting for patient-specific human leukocyte antigen (HLA) variation, and identifying the appropriate disease stage for intervention. In addition, established autoimmune disease may involve multiple autoreactive clones and tissue-specific mechanisms, potentially limiting the efficacy of targeting a single antigen [46].

## 9. Toward Precision Immune Reprogramming

The future management of autoimmune diseases is likely to move progressively away from continuous, broadly acting immunosuppression toward precision immune reprogramming. Rather than treating patients solely according to clinical phenotype, emerging approaches aim to define the molecular mechanisms driving disease in each individual and then selectively modify those pathways while preserving immune competence.

- **Biomarker-driven therapy:** Integration of transcriptomic, proteomic, genomic, and single-cell profiling may enable identification of disease endotypes and dominant inflammatory pathways before treatment initiation. Such molecular stratification could facilitate more rational selection of biologic or targeted therapies, predict treatment response, and reduce empirical trial-and-error prescribing.
- **Rational combination therapies:** Combination strategies targeting complementary pathogenic pathways may provide deeper and more durable responses than single-agent therapy, particularly in diseases characterized by biological redundancy. Future combinations may incorporate agents with different mechanisms, for example, simultaneous modulation of inflammatory cytokines, B-cell responses, and pathogenic plasma-cell compartments while carefully balancing efficacy against cumulative immunosuppression.
- **Integration with AI and multi-omics:** Artificial intelligence and machine-learning approaches are increasingly being applied to integrate high-dimensional clinical, genomic, transcriptomic, proteomic, and immunological datasets. These tools may facilitate molecular endotyping, prediction of disease trajectories and treatment responses, identification of novel therapeutic targets, and more precise patient stratification. Ultimately, integration of multi-omics with longitudinal clinical data could enable a transition from reactive treatment of established disease toward predictive and individualized immune intervention [51].

## 10. Conclusions

Antibody-based therapeutics have transformed the clinical management of autoimmune diseases, establishing targeted biologic modulation as a cornerstone of modern therapy. Despite these advances, persistent challenges including primary non-response, immunogenicity-associated secondary treatment failure, safety concerns, and biological redundancy highlight the limitations of conventional single-target approaches and underscore the need for continued therapeutic innovation. Future progress will increasingly depend on integrating next-generation antibody platforms, biomarker-driven precision medicine, and emerging cellular therapies to better match therapeutic mechanisms with individual disease biology. Ultimately, combining targeted immune modulation with antigen-specific strategies or deeper immune-resetting approaches such as CD19 CAR-T therapy may provide a path toward more durable, potentially treatment-free disease control while preserving protective host immunity.

## Figures and Tables

**Table 1:** Selected clinically established antibody-based therapeutics in autoimmune and immune-mediated inflammatory diseases.

Target Category	Drug Agent	Target Molecule	Approved Clinical Indications	Primary Mechanism of Action
TNF Inhibitors	Infliximab, adalimumab, golimumab	TNF-α	Rheumatoid Arthritis, Psoriatic Arthritis, IBD	Neutralizes TNF-α; downregulates pro-inflammatory cytokines
IL-6 Pathway	Tocilizumab, sarilumab	IL-6R/IL-6	Rheumatoid Arthritis, Giant Cell Arteritis	Blocks IL-6 receptor signaling; attenuates acute-phase response
B-Cell Depletion	Rituximab, ocrelizumab	CD20	Rheumatoid Arthritis, ANCA-associated Vasculitis, Multiple Sclerosis	Induces B-cell depletion via ADCC, CDC, and apoptosis
B-Cell Survival	Belimumab	BLyS/BAFF	Systemic Lupus Erythematosus (SLE), Lupus Nephritis	Neutralizes BAFF/BLyS, reducing autoreactive B-cell survival
Integrin Blockers	Natalizumab, vedolizumab	α4/α4β7-integrin	Multiple Sclerosis, Crohn’s Disease, Ulcerative Colitis	Blocks leukocyte adhesion and extravasation into target tissues
IL-17 blockade	Secukinumab	IL-17A	Ankylosing Spondylitis/Psoriatic Arthritis	Neutralizes IL-17A signaling
IL-23 blockade	Ustekinumab	IL-12/23	Psoriasis, Psoriatic Arthritis, Crohn’s Disease	Blocks IL-12/23 signaling
Co-stimulation blockade	Abatacept	CD80/CD86	Rheumatoid Arthritis, Psoriatic Arthritis	Fusion protein; blocks CD28 interaction, preventing T-cell activation
Type I interferon pathway	Anifrolumab	IFNAR1	Systemic Lupus Erythematosus	Blocks type I interferon receptor signaling
FcRn blockade	Efgartigimod, rozanolixizumab, nipocalimab	FcRn	Generalized Myasthenia Gravis; Chronic Inflammatory Demyelinating Polyradiculoneuropathy	Accelerates IgG catabolism, reducing circulating pathogenic autoantibodies

Abbreviations: ANCA (antineutrophil cytoplasmic antibody), BAFF (B-cell activating factor), BLyS (B-lymphocyte stimulator), IBD (inflammatory bowel disease), and IFNAR1 (interferon-alpha/beta receptor 1).

**Table 2:** Comparison of broad/targeted antibody-based therapy and antigen-specific immunotherapy in autoimmune diseases.

Feature	Broad/Targeted Antibody Therapy	Antigen-Specific Immunotherapy
Systemic Host Defense	Partially to markedly suppressed, depending on the target and mechanism	Designed to be largely preserved
Target Range	Broad immune cell populations or inflammatory cytokines	Disease-associated immune responses
Risk of Opportunistic Infection	Variable; may be moderate to high depending on the degree and type of immune suppression	Potentially lower, although this remains to be established clinically
Primary Goal	Suppression of pathogenic immune responses and disease control	Restoration of antigen-specific immune tolerance with the potential for durable treatment-free remission

## Data Availability

No new data was created or analyzed in this study. Data availability is therefore not applicable.
